# Priorities for research in child and adolescent anxiety and depression: a priority setting partnership with youth and professionals

**DOI:** 10.12688/f1000research.74205.1

**Published:** 2021-12-01

**Authors:** Brynhildur Axelsdóttir, Lise Mette Eidet, Ragnhild Thoner, Sølvi Biedilæ, Ingrid Borren, Mari Elvsåshagen, Kristine Horseng Ludvigsen, Astrid Dahlgren

**Affiliations:** 1 Regional Centre for Child and Adolescent Mental Health, Eastern and Southern Norway, Oslo, 0484, Norway

**Keywords:** Anxiety, Depression, Children, Adolescents, Research priorities, Consensus.

## Abstract

**Background:** A starting point for evaluating the effectiveness of treatments should be to identify evidence gaps. Furthermore, such evaluations should consider the perspectives of patients, clinicians and carers to ensure relevance and potentially influence future research initiatives.

**Methods:** Our approach, inspired by the James Lind Alliance methods, involved three steps. First, we performed a document analysis by identifying interventions and outcomes in two recently published overviews of systematic reviews, which summarised the effects of interventions for anxiety and depression in children and adolescents. Second, we surveyed children and adolescents with personal experiences of depression or anxiety as well as clinicians, and asked them to suggest treatments and outcomes associated with uncertainty. Finally, we facilitated a consensus process where clinicians and youth mental health patient representatives were invited to prioritise research uncertainties in separate consensus processes.

**Results: **The survey included 674 respondents who reported a total of 1267 uncertainties. Independent coding by four investigators revealed 134 suggestions for treatments of anxiety, 90 suggestions for treatments of depression, 84 for outcomes of interventions for anxiety and 71 suggestions for outcomes of interventions for depression. Two separate priority setting workshops with eight clinicians and ten youth resulted in four independent top ten priority lists.

**Conclusion:** Top ten lists of treatments and outcome domains of anxiety and depression in children and adolescents was identified by youth and clinicians. The results may influence the research agenda, and ultimately benefit patients.

## Introduction

Anxiety and depression are common mental disorders in adolescence. Anxiety is characterised by restlessness or nervousness, poor concentration, and irritability. Depression is characterised by persistent low mood, loss of interest and enjoyment. ^1^

The prevalence of anxiety and depression increases during adolescence, and the comorbidity between these diagnoses is high among young people. ^2^ Almost 10% of adolescents will meet the criteria of an anxiety disorder. ^3^ The one-year prevalence rate of adolescent depression is estimated to be 5.6%. ^4^ In Norway, the prevalence of diagnosed depression in girls 15-17 years has increased from 1.5% to 2.3% from 2010-2013. ^5^

Both anxiety and depression in adolescence are associated with functional impairment and can affect academic achievement, which may have a lifelong effect on employment. ^6^^,^^7^ According to the WHO’s Global Burden of Disease, the leading cause of years lost due to disability (YLDs) for both genders 10-24 years is unipolar depressive disorders. ^8^ The serious consequences of anxiety and depression in adolescence highlights the need for efficient interventions, and the importance of including perspectives of their own experiences.

Currently, recommended treatments for anxiety and depression are psychological therapy, pharmacotherapy, or a combination of both. ^9^^–^^11^ There are also many other treatments used for both anxiety and depression, some based on well-founded scientific research while others can be regarded as treatment uncertainties. Such uncertainties are either consequences of a lack of research, or that the research is not adequately performed and therefore the evidence is weak. ^12^ A starting point for new research on treatment uncertainties should be to identify evidence gaps, in order to shape future research priorities. ^13^^,^^14^

Research uncertainties can be identified through user involvement. The purpose of user involvement in research is to ensure that research becomes as relevant to the population in question as possible. When initiating research on treatment effects, it has not always been common practice to obtain the perspectives of patients, clinicians or carers. ^15^^,^^16^ Thus, important research questions remain unanswered, and research funding may not be used where most needed. ^17^ A recent systematic review, based on 83 studies involving 15,722 participants, demonstrated how uncommon it is to involve children and their caregivers in setting research priorities in the field of childhood chronic disease. ^16^

A recent publication by Chevance *et al*., ^18^ published in 2020, described a similar process with adult participants in an international survey, identifying outcomes for depression that matter to patients, informal caregivers, and health-care professionals. Another process of developing an Overall Paediatric Health Standard Set [OPH-SS] of outcome measures which matters to young people and their families, internationally, was also published in 2020. ^19^ The current study complements both papers, as this paper looks at both children and adolescents, as well as desired research priorities in terms of treatments, as well as outcomes.

James Lind Alliance (JLA), a non-profit making initiative, is funded by the National Institute of Health Research (NIHR) and the Medical Research Council in the UK. The purpose of the alliance is to facilitate cooperation between clinicians and patients to identify and prioritise essential research uncertainties. Their method is transparent, accountable, and systematic, and has inspired the study described in this paper. ^20^ We recently produced two overviews of systematic reviews on the effects of interventions for anxiety and depression in children and young people, respectively. ^10^^,^^11^ This left us with a momentum for inviting users and those providing mental health services to identify and prioritise research uncertainties associated with these conditions.

The objective of this study was to identify research priorities judged as additional priorities, beyond those previously identified, which may guide the research agenda moving forward.

## Methods

### Ethics

REK, Regional Committees for Medical and Health Research Ethics, Norway was contacted for approval of the project. They concluded that the project did not require their approval as there was no registered personal data. All information was collected through Nettskjema (a web-based survey system), ascertaining a high level of data security and safety.

All respondents were given information about the purpose of the study and how the results would be managed and presented and were informed that by responding to the survey, they consented to participation in the study. The questionnaire was anonymous and once submitted, the information could not be traced back to the respondent.

In the current study, we included both qualitative and quantitative methods in three stages:

1. Document analysis: identification of interventions and outcome measures used for treating children and adolescents with anxiety and depression in two previously published overviews of systematic reviews. ^10^^,^^11^
2. Mapping study (surveys): we encouraged identification by clinicians and patient representatives (children and adolescents who have, or have had, anxiety or depression) of additional priorities outside of those previously identified.
3. Consensus process: prioritisation of research uncertainties by clinicians and patient representatives.

Our approach was inspired by a method developed by the JLA. ^20^ The method involves patients and clinicians in suggesting research priorities. Their input is given equal weight. The method is designed to raise awareness of important evidence gaps, with the potential of influencing new research initiatives. ^15^

**Figure 1.**
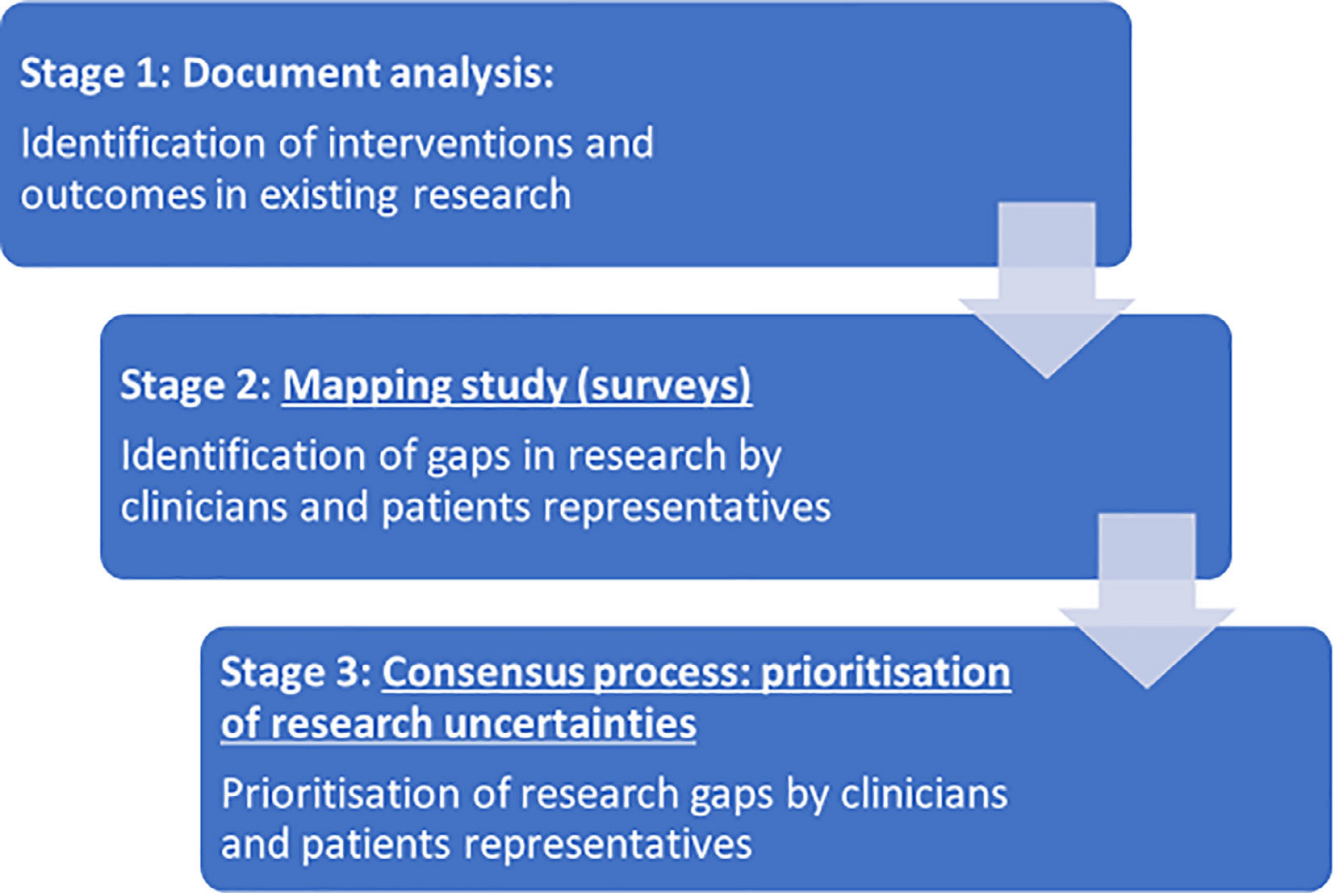
Flow chart of the method process.

The stages of the prioritisation process are outlined in Figure 1.

### Document analysis: identification of interventions and outcomes in existing research

In two recently published overviews of systematic reviews, we have summarised the effects of interventions for anxiety and depression in children and adolescents. ^10^^,^^11^ Although these publications are in Norwegian, the methodology of the review process have been published in registered protocols and is available in English through the PROSPERO database; CRD42020159883 (depression) and CRD42020159884 (anxiety). To provide context to this paper, we briefly describe the inclusion criteria and search strategy of the reviews here. Both overviews adhered to the PRISMA guidelines ^21^ and to the following inclusion criteria:

Publications: Systematic reviews published 2012 and later, fulfilling the DARE-criteria.

Language: English, Norwegian, Danish, or Swedish.

Participants: Children and adolescents under the age of 18, with or without an identified risk of developing mental health problems or those who have already developed these problems.

Intervention: Any intervention aimed at preventing or reducing mental health problems or welfare interventions, including psychological therapy, pharmaceutical interventions, psychosocial interventions etc.

Comparison: Other relevant interventions, treatment as usual (TAU), no treatment or wait list.

Outcomes: All outcomes of mental health problems and child welfare evaluated in children and adolescents, including other health outcomes, quality of life, function, use of health care, attitudes and adverse effects of interventions.

The search for reviews that were included in these two overviews was largely based on the IN SUM database and was performed in April 2018, with an updated search in December 2018. IN SUM is a recently developed database of systematic reviews of the effects of interventions relevant to children and young people’s mental health and welfare. The database indexes systematic reviews from the following databases: Cochrane Database of Systematic Reviews, Campbell Library, PsycINFO, Medline, Embase, Web of Science, Database of Abstracts of Reviews of Effects (DARE) and Evidence-Based Mental Health. IN SUM is continuously updated monthly with the latest systematic reviews. In addition to IN SUM, we hand searched the websites of the Norwegian Institute of Public Health, the Swedish Agency for Health Technology Assessment and Assessment of Social Services, the Danish Health Authority for Systematic Reviews and the National Institute for Health and Care Excellence for evidence-based guidelines, UK. For complete search strategies see *Extended data.* ^28^

The first author (BA) extracted all interventions and outcomes reported in these two overviews in a simple document analysis and second author (AD) double-checked the extraction.

### Mapping study (survey): identification of uncertainties in research

We created three surveys, each including four questions asking the respondents to report what treatments and outcomes ought to be topics for research, in their opinion. For each question, the recipients were presented with a list of the treatments and the outcomes already addressed in existing research (see Table 1, Table 2), based on the two overviews of reviews. ^10^^,^^11^ The three surveys were distributed to clinicians and users as an electronic questionnaire via Nettskjema.

**Table 1.** List of treatments and outcomes of anxiety based on existing research. **

**Treatments for anxiety**
Psychoeducation *
Cognitive behavioural therapy
Family therapy
Psychodynamic therapy *
Mindfulness
Pharmacological therapy *
**Outcomes in anxiety**
Symptoms of anxiety
Less self-harm (including suicide) *
Treatment satisfaction *
Daily functioning
Drop-out from treatment
Adverse events *

* The quality of the evidence is graded as low or very low.

** Treatments and outcomes in 2018, more treatments and outcomes are described in the 2021 update.

**Table 2.** List of treatments and outcomes of depression based on existing research **

**Treatments for depression**
Psychoeducation *
Cognitive behavioural therapy
Interpersonal therapy *
Dialectical therapy *
Behavioural activation *
Psychodynamic therapy *
Family therapy *
Mindfulness *
Play therapy *
Art therapy *
Exercise/Physical activity *
Pharmacotherapy *
Electroconvulsive therapy (ECT) *
Repetitive transcranial magnetic stimulation (rTMS) *
**Outcomes in depression**
Symptoms of depression
Anxiety *
Mania *
Self-harm (including suicide) *
Treatment satisfaction *
Function *
Drop-out from treatment *
Adverse events *

* The quality of the evidence is graded as low or very low.

** Treatments and outcomes in 2018, more treatments and outcomes are described in the 2020 update.

The survey questions had an open answer option (see *Extended data* ^28^). Respondents can be unfamiliar with research, and we therefore considered it more appropriate to let respondents formulate their need for research in their own words. ^20^ The purpose of the surveys was to collect suggestions for research uncertainties, consequently, the sample did not need to be representative. ^20^ Instead, we used convenience sampling to recruit the participants. Anyone living in Norway with experience and understanding of living with anxiety or depression was eligible to participate in the identification of uncertainties. This included children and adolescents with anxiety and/or depression, carers, family members and friends. Also, healthcare, and social care professionals who had worked with children and adolescents living with the conditions were eligible. We strived to ensure that professionals working in different levels in health and welfare services were represented, as well as users. No demographic data were collected as it is not a part of later analysis in priority setting partnerships. The JLA guidebook recommends that the purpose of collecting demographic information from respondents is for this purpose only. ^20^

The first survey was sent on 22 ^nd^ February 2019, to our institution's contacts working with children and young people's mental health in the municipalities (Eastern and Southern Norway), including employees in child welfare institutions/orphanages, special education teachers working in schools, child welfare services, child welfare guards, family protection offices, refugee and immigration departments.

The second survey was distributed on 19 ^th^ March 2019, to professionals working in the specialist mental health service for children and adolescents. These were also contacted through our networks. In addition, we recruited respondents in collaboration with the Norwegian Association for Children and Young People’s Mental Health (NBUP) and from our institution’s newsletter.

The third survey was distributed on 25 ^th^ April 2019, to children and adolescents having personal experiences with depression and/or anxiety, as well as to their carers, in collaboration with the Norwegian organisation for youth mental health, Mental Helse Ungdom (MHU). We also sought to recruit respondents through social media platforms of our institution, e.g., Facebook and Instagram. We posted a link of the survey on the platforms 2 ^nd^ August 2019, with an invite to eligible participants to complete the survey.

### Content analysis

The interventions and outcomes suggested by the respondents were coded independently by at least two investigators (IB, SB, LME and BA). This part of the process is both interpretative and subjective. Duplicates and similar submissions were combined to a common suggestion. Combining submissions can greatly reduce the volume of data in the process of finalising a top ten list. ^20^ Based on this analysis we created four “master-lists” including all suggestions for:

1) interventions for anxiety
2) interventions for depression
3) outcomes of interventions for anxiety
4) outcomes of interventions for depression

### Consensus process: prioritisation of research uncertainties

*Preparations for the consensus process*

The next step was to prepare for the consensus process, where selected professionals and users were asked to prioritise the suggested research uncertainties. There is no gold standard for conducting a consensus process. However, group composition can have an impact and may lead to different judgements. ^22^

A multi-disciplinary team of professionals were recruited through our networks through convenience sampling. We received help recruiting clinicians from a local child and adolescent psychiatric outpatient clinic. Our contact person there, reached out via e-mail on 21 ^st^ August 2019, to clinicians with a request to participate in the consensus process. The criteria were clinicians who work, or have worked, with children and adolescents with anxiety or depression. A variety of professionals from different backgrounds and working at different levels of health and welfare services (such as psychologists, psychiatrists, physiotherapists, nurses, educators, and health nurses) came forward. Seven clinicians from the specialist mental health services and four from the municipal health services accepted the invitation to participate. For recruitment of user representatives, we contacted the Assistant General Secretary of MHU. She reached out via e-mail on 15 ^th^ September 2019, to their members of staff and youth with experience of the conditions, and twelve participants accepted the invitation.

Once recruited, we received contact information of 10 participants proposed by the assistant general secretary of the organisation on October 10 ^th^,2019. We emailed the four lists with the suggested interventions and outcomes for anxiety and depression, respectively to the participants. They were individually asked to put the suggestions in ranked order, by selecting only 10 options that were assigned 1 point each. For the three most important options we asked them to assign these 2 points. This resulted in the first drafts of prioritised lists of interventions, and outcomes of interventions, for anxiety and depression.

The results from this pre-prioritisation were summarised by two members of the research team (AD and BA), and four lists were created with the highest-ranking suggestions. According to the JLA method, uncertainties must be checked and verified as true uncertainties before prioritisation can begin. ^20^ The two overviews of systematic reviews documented which treatments and outcomes that lacked or had weak scientific evidence. ^10^^,^^11^ The participants of the workshops were made aware of this before conducting the interim prioritisation, also enabling them to prioritise among those.

*The workshops*

For practical reasons, it was not possible to host a shared workshop for professionals and users. Instead, separate workshops were held.

When conducting consensus processes, the criteria for establishing priorities should be applied using a systematic and transparent process. ^22^ Furthermore, group discussions should follow some basic rules that the participants have chosen jointly. Participants should listen to each other and show respect for each other’s ideas. ^20^

According to the JLA, it is recommended to base the consensus process on the Nominal Group Technique, which we applied for both workshops. ^20^ This approach is characterised as a structured method for group brainstorming, encouraging discussion and facilitating agreement on the relative importance of issues in question. The process should be led by someone who is not part of the project group, and according to JLA, who has no research background. The person will, therefore, have a more neutral role in the process. It is essential that the entire process has openness and justice as guiding principles. ^20^ For this study, we invited an experienced expert in consensus processes to facilitate and host the workshops (RT), the rest of the team played the part of silent observers and handled all practical needs (LME, SB, AD, and BA).

The first workshop was held at our organisation’s location in Oslo, Norway on 26 ^th^ September 2019, from 9:00 am to 3:00 pm. Three members of the project group attended the workshop in addition to the consensus host (LME, RT, AD, and BA). Eight out of 11 clinicians were able to participate in the workshop: psychologists, special educators, clinical social workers, and a physician. Three clinicians were unable to attend for various reasons such as sickness etc.

For the second workshop, we recruited youth from MHU. The workshop took place in their location on 11 ^th^ November 2019, from 9:00 am to 3:00 pm and was administrated in the same way as the workshop with the clinicians. Ten out of 12 invited youth were able to participate in the priority setting, and three members of the project group facilitated the workshop (RT, SB and LME). Two participants were unable to attend. 

After formal introductions and light refreshments, the participants received an introduction for one hour, to the principles of research, systematic reviews, and evidence-based practice. They were also informed about the purpose and agenda of the day. Thereafter, the participants were divided into small groups based on their professional background, age and in the workshop with the youth, earlier experience with anxiety and/or depression. For each topic, the participants were then mixed in different groups with at least three participants in each group. This part of the workshops lasted for four hours with a half an hour lunch break.

The groups were assigned the task of selecting 10 options and prioritising these for each topic. The groups worked independently but were facilitated by the host when necessary. Other members of the project group were silent observers, taking notes. At the workshop with the professionals, the host used images of children and adolescents with depression and anxiety during this process, as a reminder of the perspectives of the target group involved.

The final hour of the workshops included individual prioritising. All four lists were entered into a voting app by one of the members of the project group and each participant was asked to anonymously rank the final top ten priorities per list. This resulted in four top ten lists of priorities ranked in order by their perceived importance [see *Underlying data* ^28^].

## Results

### Summary of existing research

The results of the document analysis were collated and made into 4 lists. In the surveys, the respondents were presented with these lists (see Table 1 and Table 2). Note that for several of these treatments and outcomes, the quality of the evidence is graded as low or very low (marked with * in the tables). Therefore, these could still be suggested as research uncertainties.

### Results of the surveys: identified research uncertainties by clinicians and patient representatives

Overall, 674 respondents submitted a total of 1267 research suggestions in the three surveys. After content analysis, 379 unique suggestions (134 treatments for anxiety, 90 treatments for depression, 84 outcomes for anxiety and 71 outcomes for depression), were sent for ranking via e-mail to the clinicians and youth participating in the workshops.

**Figure 2.**
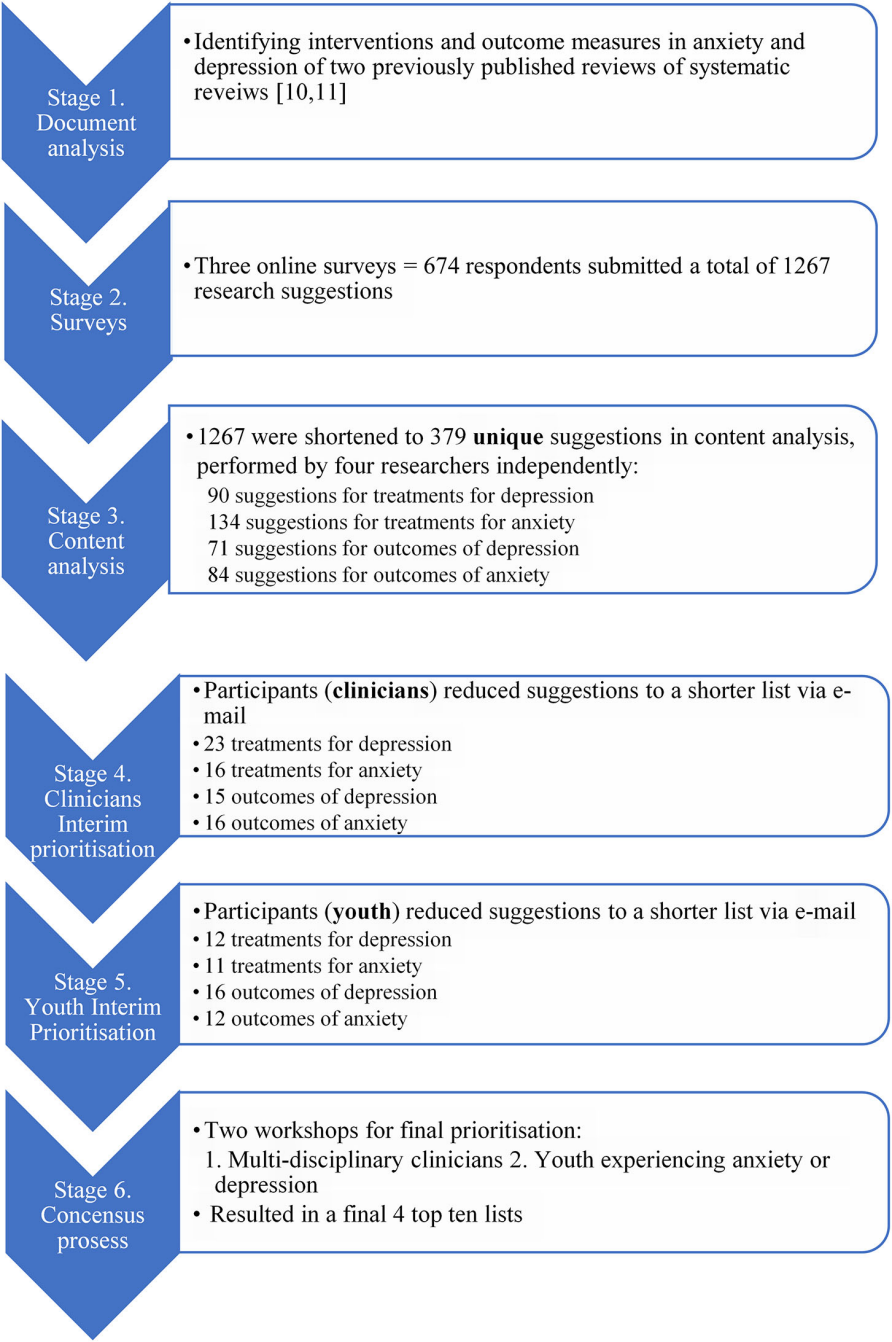
Flow chart of full process with results.

In response, the clinicians ranked and shortened the list to 70 suggestions. The youth ranked and shortened it to 51 suggestions. For full detail of the results of the process see Figure 2.

### Prioritisation of research uncertainties

Eight clinicians participated in the first workshop: psychologists, special educators, clinical social workers, and a physician. Two of the clinicians worked in the mental health services in the municipalities, and the six others worked in the specialist mental health service for children and adolescents.

The 10 youth participants from MHU participated in the second workshop. See detailed results of the process in Figure 2 and the final results of the workshops priority setting in Tables 3, 4, 5 and 6.

**Table 3.** Prioritised treatments for anxiety.

Treatments for anxiety prioritised by clinicians	Treatments for anxiety prioritised by youth
1. Family therapy [not based on cognitive behavioural therapy]	1. Parent-based interventions
2. Increased cooperation between mental health services and schools	2. Interventions for forming relationships
3. Further treatment for “none-responders”	3. Resilience groups
4. School-based interventions	4. Coping with life strategies [as a school subject]
5. Parent-based interventions	5. Increased cooperation between mental health services and schools
6. Exposure therapy	6. Exposure therapy
7. Psychoeducation	7. Multi-disciplinary cooperation
8. Attachment disorders	8. Therapy for transgender persons
9. Emotion-focused parent training	9. Circle of security
10. Window of tolerance	10. Recreation therapy

**Table 4.** Prioritised outcomes of interventions for anxiety.

Outcomes of interventions for anxiety prioritised by clinicians	Outcomes of interventions for anxiety prioritised by youth
1. Friends and social activities	1. Resilience
2. Family functioning	2. Daily life functioning
3. Quality of life	3. Treatment satisfaction
4. Evasive behaviour	4. Trust in other people
5. School functioning	5. Family functioning
6. Long term follow-up	6. Professional functioning
7. Emotion regulation	7. Self-harm (suicide)
8. Adverse events	8. Long term follow-up
9. Treatment satisfaction	9. Help-seeking behaviour
10. Sleep	10. Physical activity

**Table 5.** Prioritised treatments for depression.

Treatments for depression prioritised by clinicians	Treatments for depression prioritised by youth
1. Family therapy	1. Easy access to treatment
2. Parent-based interventions	2. Forming relationship
3. Group treatment	3. Multi-disciplinary cooperation
4. School-based interventions	4. Sleep therapy
5. Systemic practice	5. School-based prevention programmes
6. E-therapy	6. E-therapy
7. Recreational activities	7. Group treatment
8. Light, sleep and nutritional therapies	8. Interventions providing access to school psychologist
9. Play therapy	9. Training of health personnel
10. Circle of security	10. Friendship groups

**Table 6.** Prioritised outcomes of interventions for depression.

Outcomes of interventions for depression by clinicians	Outcomes of interventions for depression by youth
1. Friends and social activities	1. Daily life functioning
2. Quality of life	2. Faith in oneself and the future
3. Family functioning	3. Professional functioning
4. School functioning	4. Identity
5. Emotion regulation	5. Life skills
6. Adverse events	6. Self-harm (suicide included)
7. Drop-out of treatments	7. Emotion regulation
8. User involvement	8. Sexuality
9. Daily life functioning	9. Number of emergency inquiries
10. Attachment	10. Long term follow-up

## Discussion

This study has demonstrated essential research priorities in terms of treatments that should be evaluated and outcomes that should be measured according to youth and clinicians. The top ten lists reflect both similarities and differences in what is considered important by the clinicians and the youth.

Clinicians ranked family and parent-based interventions as their top priority for both lists of treatments (anxiety and depression). Youth also ranked family and parent-based interventions as their top priority for treatments of anxiety. Functioning in daily life, and in the family are amongst the top ten treatment priorities by both groups. Other common priorities important to both clinicians and youth are increased cooperation between mental health services and schools, and multi-disciplinary cooperation.

Top priority for depression treatment among the adolescents, were easy access to treatment. The clinicians also emphasize increased cooperation between mental health services and schools, as well as group treatment and school-based interventions. Thus, the clinicians seem to focus on strengthening the environment around the youth to a greater extent than the adolescents do. School-based therapies, school functioning and access to a school psychologist are also similar priorities. The youth seem, however, to display a greater need for interventions for forming relationships, resilience groups, and life coping strategies, which is not mentioned at all in the clinicians’ list.

A unique priority suggested by the youth is therapy for transgender people, specifically regarding anxiety. This may demonstrate a difference between generations regarding the focus on gender identity and the need to cope with such issues.

On the lists of outcomes of interventions for both conditions, functioning in daily life, in the family, and at work were ranked very high by both the clinicians and the youth, as well as friends and social activities. Other important common suggestions are long-term follow-up of interventions, treatment satisfaction and user involvement. However, it is worth noting that the outcomes most important for the adolescents, for both anxiety and depression, were highly subjective/internal outcomes like resilience, faith in oneself, life skills, identity, daily life functioning and trust in other people. In contrast, the clinicians ranked friends and social activities on top of both lists, while this suggestion was not found on the adolescent’s lists. Thus, the clinicians seem to view the context the youth is in as more important than the youths do themselves, who to a greater extent emphasize personal coping skills, like faith in oneself and resilience. This difference may possibly tell us that contextual factors (friends, school or dropping out of school) are regarded less important for individuals struggling with mental health challenges, and that inner personal growth and mastery are key factors for these young people. The clinicians may, on the other hand, have been thinking more in terms of outcomes known to be preventive factors (like friendship and social structures). ^23^

Clinicians rated adverse events as important for both conditions. The lack of research of unwanted effects of treatments for depression in children and adolescents has recently been demonstrated in a mapping of systematic reviews. ^24^ Both the clinician’s views and Eidet’s article ^24^ point to the need for more research, and thus address adverse events in these treatment groups as an important evidence gap.

### Strength and limitations

This study builds on rigorous qualitative and quantitative methods, including two extensive systematic reviews on the effects of treatments for anxiety and depression. To our knowledge this is also the first mapping study in Norway exploring research uncertainties related to treatments and associated outcomes for anxiety and depression.

The current study is in line with evidence-based practice as it is defined as ‘The conscientious, explicit and judicious use of current best evidence in making decisions about the care of the individual patient’. ^25^ Evidence-based practice highlights the consideration of the patient’s opinions in choice of treatment (alongside clinical opinions and research-based methods), and the current project contributes along these lines also, by letting patients voice their concerns regarding research gaps. We have integrated the best research evidence and involved clinical expertise both in the surveys and the workshop with clinicians. Furthermore, we have included the personal and unique values of the patients. All of these should be a part in any decision-making process concerning research and treatments for children and adolescents.

There has been increasing attention to patient-reported outcomes during recent years. Outcomes should be relevant and important to both patients, caregivers, health care professionals and other stakeholders making decisions about health care. ^26^^,^^27^ For discovering what outcomes are important to patients and health care professionals, consensus processes, as demonstrated in this study, are vital. This study is especially important because we succeeded in including the views of young people, considering how rare patient and family engagement are in research priority setting. ^16^

The importance of user involvement is demonstrated in feedback from participants in both workshops:

“ *It feels very meaningful to be able to contribute to this project on behalf of all the patients I have been in contact with*”.

“ *Children and adolescents should always be involved in decision-making, not just clinicians*”.

The limitation of consensus processes should be acknowledged. The current priorities are based on individual’s or groups’ point of views and their subjective opinions. We might, in our consensus process with a different pool of people in a different situation, reach a different result. ^20^ However, involving people together in a quality discussion to reach genuine consensus is of great value, as it represents an important contribution to the debate on research priorities. Bringing people together in a workshop enables them to exchange knowledge and information and make decisions in their meetings with the health services, based on a wider set of experiences.

Initially we intended to host only one priority setting workshop with both clinicians and the youth as recommended by JLA, however we were unable to find an appropriate date suitable for both groups. Although hosting a shared workshop would have had several benefits, we also found it useful to keep the groups separated. We were able to avoid challenges, such as ensuring the choice of participants being balanced, avoiding domination by one person, and reaching consensus when there may have been disagreement. The two separate processes allowed us to compare the results of professionals and the youth. It also provided a safe zone for professionals and the youth, where especially the latter could speak more freely and perhaps avoid feeling ‘led’ to conclusions by clinicians whom they perhaps could see as authority figures with more experience than themselves. However, keeping the groups separate meant that we also missed the opportunity of cross-fertilization of ideas and nuancing of perspectives, that mixing professionals and users may have contributed to.

## Conclusion

We have demonstrated the possibility to develop an agreed four top ten lists of research priorities for anxiety and depression in children and adolescents, with contribution from youth experiencing anxiety or depression as well as clinicians. The perspectives from their individual lists, have the possibility to influence the research agenda according to the needs and opinions of both clinicians and the patients themselves.

## Data availability

### Underlying data

Harvard Dataverse: Priorities for research in child and adolescent anxiety and depression: a priority setting partnership with youth and professionals https://doi.org/10.7910/DVN/UQPYVT. ^28^

This project contains the following underlying data:

• Coding_priorities from participants_Clinicians_final_25.09.2019.tab
• Coding_Priorities_Adolescents_Final_07.11.2019.tab

### Extended data

Harvard Dataverse: Priorities for research in child and adolescent anxiety and depression: a priority setting partnership with youth and professionals https://doi.org/10.7910/DVN/UQPYVT. ^28^

This project contains the following extended data:

• Tables 3-6 (in Norwegian, pdf.)
• Appendix I (Copy of survey no.1, no.2. and no.3.)
• IN SUM Search strategies_2021.pdf

Data are available under the terms of the Creative Commons Zero “No rights reserved” data waiver ( CC0 1.0 Public domain dedication).
