# Supplementary material for: Research in child and adolescent anxiety and depression: treatment uncertainties prioritised by youth and professionals
Source: F1000Res. 2022 May 3;10:1221. Originally published 2021 Dec 1. [Version 2] doi: 10.12688/f1000research.74205.2 (PMC9377379; doi:10.12688/f1000research.74205.2)
Supplement: Peer review history

# Peer review: Reviewer response for version 2

**DOI:** 10.5256/f1000research.133634.r136572

**Published:** 2022-07-22

**Authors:** Judith Borghouts

**Affiliations:** 1 Department of Medicine, University of California Irvine, Irvine, CA, USA

I have looked at the authors’ responses and am satisfied with changes made.

Is the work clearly and accurately presented and does it cite the current literature?

No

If applicable, is the statistical analysis and its interpretation appropriate?

Not applicable

Are all the source data underlying the results available to ensure full reproducibility?

Yes

Is the study design appropriate and is the work technically sound?

Partly

Are the conclusions drawn adequately supported by the results?

Partly

Are sufficient details of methods and analysis provided to allow replication by others?

Yes

Reviewer Expertise:

Academic researcher in Digital Mental Health and Human-Computer Interaction with 10 years of experience in quantitative and qualitative research.

I confirm that I have read this submission and believe that I have an appropriate level of expertise to confirm that it is of an acceptable scientific standard.

# Peer review: Reviewer response for version 2

**DOI:** 10.5256/f1000research.133634.r136571

**Published:** 2022-05-12

**Authors:** Kristina Staley

**Affiliations:** 1 TwoCan Associates, Ross-on-Wye, UK

I have looked at the authors’ responses and changes and am satisfied that the issues I raised have been addressed. I am happy to recommend the revised version for indexing.

Is the work clearly and accurately presented and does it cite the current literature?

Yes

If applicable, is the statistical analysis and its interpretation appropriate?

I cannot comment. A qualified statistician is required.

Are all the source data underlying the results available to ensure full reproducibility?

Yes

Is the study design appropriate and is the work technically sound?

Partly

Are the conclusions drawn adequately supported by the results?

Partly

Are sufficient details of methods and analysis provided to allow replication by others?

Yes

Reviewer Expertise:

NA

I confirm that I have read this submission and believe that I have an appropriate level of expertise to confirm that it is of an acceptable scientific standard.

# Peer review: Reviewer response for version 1

**DOI:** 10.5256/f1000research.77933.r126468

**Published:** 2022-03-23

**Authors:** Judith Borghouts

**Affiliations:** 1 Department of Medicine, University of California Irvine, Irvine, CA, USA

This paper describes an approach, inspired by James Lind Alliance (JLA) methods, to identify research priorities in child and adolescent anxiety and depression treatments. Strengths of the paper are the detailed descriptions of the methods used. I also appreciate the authors making the data available.

 It was however quite unclear what the paper is trying to contribute, as the problem, objective and results do not seem aligned. The paper starts by highlighting the problem of treatment uncertainties, and that some treatments lack scientific evidence. The introduction then states that the objective of the study was to identify research priorities, which seems different from treatment uncertainties. Finally, it presents results of what types of treatments clinicians and youth would like to see. If this all relates to the same thing, the paper should do a better job explaining how these are all connected.

 Related to my point above, the key terms are not well-defined. The abstract mentions treatment uncertainties but it is unclear what this is. It becomes a little bit clearer through examples given in the introduction (“uncertainties are either consequences of a lack of research, or the research is not adequately performed”), but it is then not clear how you ‘prioritize’ uncertainties? Do the authors mean which type of treatments should be given priority in future research? Furthermore, Table 4 and 6 mention the term ‘outcomes’, which in the context of treatment usually means treatment outcomes, such as measurable health symptoms. A number of the outcomes in these tables do not seem to be outcomes in the traditional sense; for example, how is ‘friends and social activities’ an outcome? Is this somehow related to social connectedness? The paper is currently lacking a clear explanation of all of these terms, concepts and how they relate to one another.

 Lastly, if the objective was to identify research priorities, it was not clear to me why non-researchers were asked to identify uncertainties. As the paper states, respondents can be unfamiliar with research and may not be equipped to prioritize research. It seems that the paper instead collected a stakeholder perspective of important considerations in adolescent treatment for anxiety and depression, which is still important, but is not reflected in the paper’s objective at all.

 I recommend the authors to clearly define the key concepts, clarify the problem, aim of the study, how the results address this problem and aim, and make this consistent throughout the paper.

Is the work clearly and accurately presented and does it cite the current literature?

No

If applicable, is the statistical analysis and its interpretation appropriate?

Not applicable

Are all the source data underlying the results available to ensure full reproducibility?

Yes

Is the study design appropriate and is the work technically sound?

Partly

Are the conclusions drawn adequately supported by the results?

Partly

Are sufficient details of methods and analysis provided to allow replication by others?

Yes

Reviewer Expertise:

Academic researcher in Digital Mental Health and Human-Computer Interaction with 10 years of experience in quantitative and qualitative research.

I confirm that I have read this submission and believe that I have an appropriate level of expertise to state that I do not consider it to be of an acceptable scientific standard, for reasons outlined above.

# Response

**Published:** 2022-05-04

**Authors:** Brynhildur Axelsdottir

**Affiliations:** Regional Centre for Child and Adolescent Mental Health, Eastern and Southern Norway (RBUP), Norway

1.This paper describes an approach, inspired by James Lind Alliance (JLA) methods, to identify research priorities in child and adolescent anxiety and depression treatments. Strengths of the paper are the detailed descriptions of the methods used. I also appreciate the authors making the data available.

 It was however quite unclear what the paper is trying to contribute, as the problem, objective and results do not seem aligned. The paper starts by highlighting the problem of treatment uncertainties, and that some treatments lack scientific evidence. The introduction then states that the objective of the study was to identify research priorities, which seems different from treatment uncertainties. Finally, it presents results of what types of treatments clinicians and youth would like to see. If this all relates to the same thing, the paper should do a better job explaining how these are all connected.

 Related to my point above, the key terms are not well-defined. The abstract mentions treatment uncertainties but it is unclear what this is. It becomes a little bit clearer through examples given in the introduction (“uncertainties are either consequences of a lack of research, or the research is not adequately performed”), but it is then not clear how you ‘prioritize’ uncertainties? Do the authors mean which type of treatments should be given priority in future research? Furthermore, Table 4 and 6 mention the term ‘outcomes’, which in the context of treatment usually means treatment outcomes, such as measurable health symptoms. A number of the outcomes in these tables do not seem to be outcomes in the traditional sense; for example, how is ‘friends and social activities’ an outcome? Is this somehow related to social connectedness? The paper is currently lacking a clear explanation of all of these terms, concepts and how they relate to one another.

**Response: We see the reviewers point; however, we see this as the one influencing the other. What we hope with our process is that identified treatment uncertainties (treatments that lack scientific evidence) should become priorities in future research. Research priorities should be based on research uncertainties established by systematic reviews of the existing evidence. We have added a sentence to make this more explicit.**

**By “treatment” we refer  to any action or intervention used to change an aspect of a young person’s mental health, that being medicines or school-based interventions. Such treatments may also have an impact on other aspects of the young person’s life that may be important to consider in research. As we state in the paper, the outcomes found to be important to evaluate in research by researchers often differs from those of providers and patients. Thus, in many cases effects on outcomes important to patients and providers are unknown. This study tries to address this issue. Such outcomes may include a person’s ability to participate in social activities and so on.**

**Our aim was to enable the participants to suggest and prioritise preferred treatments and outcomes and thus highlight the needs of users and clinicians in hope that these needs could be met in future studies. The lists of priorities are the outcome of this whole process, presenting the interventions and outcomes that the involved groups would like to see in future studies. We acknowledge that this link may have not been sufficiently elaborated on and have therefore inserted some sentences that may help clarify the link between these stages of the process in the introduction and the discussion. The objectives have also been rephrased and hopefully appear clearer.**

 2. Lastly, if the objective was to identify research priorities, it was not clear to me why non-researchers were asked to identify uncertainties. As the paper states, respondents can be unfamiliar with research and may not be equipped to prioritize research. It seems that the paper instead collected a stakeholder perspective of important considerations in adolescent treatment for anxiety and depression, which is still important, but is not reflected in the paper’s objective at all.

**Response: The idea here is to involve the perspectives of the patients involved and the professionals that treat them. They have unique insights in their needs, which may deviate from the priorities of a researcher. Further, user involvement is one of the main principles of the JLA guidebook, which have partly inspired us in conducting this study. The JLA initiative was established to bring both patients, carers and clinicians together in priority setting partnerships. This ensures shared decision-making processes, which is a cornerstone of evidence-based practice.**

 3.I recommend the authors to clearly define the key concepts, clarify the problem, aim of the study, how the results address this problem and aim, and make this consistent throughout the paper.

**Response: We have, based on the reviewers’ responses, rephrased the aim, and sought to make the objective clearer. We believe that our responses to other remarks from the reviewers may also make the paper more accessible. In the introduction, we have described some of the key concepts for clarification.**

**We would like to thank the reviewers for their valuable comments.**

# Peer review: Reviewer response for version 1

**DOI:** 10.5256/f1000research.77933.r124299

**Published:** 2022-02-22

**Authors:** Kristina Staley

**Affiliations:** 1 TwoCan Associates, Ross-on-Wye, UK

This paper reports on a priority setting exercise which has drawn on the JLA approach but has changed so far from it that I question whether to make the links is appropriate. For example I'd challenge the use of the term priority setting partnership in the title.

 The approach in this paper differs from the JLA process in two main ways:

1. Categorising the uncertainties collected via survey of young people and professionals. In a JLA process the Steering Group, a mix of professionals and affected patients/carers, are heavily involved in interpreting the responses to generate a list of uncertainties using phrasing and language that summarise the responses. The aim is always to stay faithful to the original responses. In this paper the researchers have drawn out interventions and outcomes as separate lists - not whole questions. I do not understand the rationale for this and would like a clearer explanation in the article. As they have identified, the language used and the priority given to different ways of understanding the issues makes it difficult to combine the youth and professionals' priority lists of interventions/outcomes. In the JLA process, this is done in the partnership of the Steering Group to reach a shared agreement of the list of topics to be prioritised, a shared understanding of what these mean so that people from all perspectives can understand and prioritise the shared list.
  Furthermore, I'd like the authors to comment on how the prioritised lists of interventions and outcomes might be used to shape future research.
2. The final workshop - it is essential that all parties come together and reach a shared agreement of the Top Ten. It would seem important to find a date for such a meeting that all could attend rather than have separate meetings. And for the group discussion to inform the prioritised list rather than individuals voting on an app. 

So in general there seems to have been limited *shared* decision-making at each of the stages of this process which makes me question whether this was genuinely a partnership or actually different groups prioritising topics separately. This is what makes it very different to the JLA process.

 The outputs are quite distinct from those of a JLA process - so I suggest the authors refer to the JLA perhaps once, and instead describe their own process and the rationale for how they have approached it, what they expect the impact to be, and their perceived value of their outputs. 

 Different does not mean better or worse - this is a different process to the JLA and may have strengths or weaknesses as a result. Perhaps these could be explored in the article. The JLA is not a set of methods, but the principles and values that underpin partnership working are absolutely key to it and these are not described in the approach in this paper and I therefore recommend that the suggestions that this process is linked to the JLA approach are reduced.

Is the work clearly and accurately presented and does it cite the current literature?

Yes

If applicable, is the statistical analysis and its interpretation appropriate?

I cannot comment. A qualified statistician is required.

Are all the source data underlying the results available to ensure full reproducibility?

Yes

Is the study design appropriate and is the work technically sound?

Partly

Are the conclusions drawn adequately supported by the results?

Partly

Are sufficient details of methods and analysis provided to allow replication by others?

Yes

Reviewer Expertise:

I have worked on over a dozen JLA PSPs as an Information Specialist and have worked in the field of patient and carer involvement in research for over 20 years

I confirm that I have read this submission and believe that I have an appropriate level of expertise to state that I do not consider it to be of an acceptable scientific standard, for reasons outlined above.

# Response

**Published:** 2022-05-04

**Authors:** Brynhildur Axelsdottir

**Affiliations:** Regional Centre for Child and Adolescent Mental Health, Eastern and Southern Norway (RBUP), Norway

1.This paper reports on a priority setting exercise which has drawn on the JLA approach but has changed so far from it that I question whether to make the links is appropriate. For example I'd challenge the use of the term priority setting partnership in the title.

 The approach in this paper differs from the JLA process in two main ways:

 Categorising the uncertainties collected via survey of young people and professionals. In a JLA process the Steering Group, a mix of professionals and affected patients/carers, are heavily involved in interpreting the responses to generate a list of uncertainties using phrasing and language that summarise the responses. The aim is always to stay faithful to the original responses. In this paper the researchers have drawn out interventions and outcomes as separate lists - not whole questions. I do not understand the rationale for this and would like a clearer explanation in the article. As they have identified, the language used and the priority given to different ways of understanding the issues makes it difficult to combine the youth and professionals' priority lists of interventions/outcomes. In the JLA process, this is done in the partnership of the Steering Group to reach a shared agreement of the list of topics to be prioritised, a shared understanding of what these mean so that people from all perspectives can understand and prioritise the shared list.

 Furthermore, I'd like the authors to comment on how the prioritised lists of interventions and outcomes might be used to shape future research.

**Response: We thank the reviewer for pointing this out and we acknowledge the differences of our study and the James Lind Alliance framework. We have therefore changed the title of the article. In addition, we have elaborated on these differences in methods and discussion.**

**As to the comment on how the** **prioritised lists of interventions and outcomes might be used to shape future research, we strongly believe that researchers can be inspired to see what interventions lack evidence (based on evidence gaps identified by the overviews of systematic reviews) as well as what outcomes should be measured when designing new studies on these subjects, based on the participants’ priorities. To highlight the desired interventions and outcomes of users and clinicians may hopefully bring awareness to researchers regarding the needs of these groups – potentially enhancing shared decision-making in future studies.**

 2. The final workshop - it is essential that all parties come together and reach a shared agreement of the Top Ten. It would seem important to find a date for such a meeting that all could attend rather than have separate meetings. And for the group discussion to inform the prioritised list rather than individuals voting on an app.

 So in general there seems to have been limited shared decision-making at each of the stages of this process which makes me question whether this was genuinely a partnership or actually different groups prioritising topics separately. This is what makes it very different to the JLA process.

**Response: There is no gold standard to priority setting of research uncertainties. JLA has however developed an extensive experience and evidence base in this area which has inspired our efforts.**

**We acknowledge that our approach differs from that of the JLA. We have revised our manuscript to make this clearer and have made it explicit which of the methodological choices recommended by JLA we have applied. We have also added a paragraph to the discussion about the potential limitations and strengths of the choices we made.**

**As the reviewer points out, the JLA is not a set of methods but suggests some principles and values that should be considered. The experts and patients taking part in our study were not able to meet in the same day for the consensus workshop, and thus our process resulted in two separate sets of priority lists. Although the resulting lists were not created in a partnership of patients and providers, the results of these two consensus processes provides the opportunity to compare the differences in priorities by patients and providers. This may have brought additional – and potentially valuable – information and possibly cover more evidence gaps.**

**Even though our process differs from that of JLA, we have used methods of high quality, including basing our process on high-quality systematic reviews, including both qualitative and quantitative feedback from experts and patients, and applying a recognized consensus-process methodology. We believe that the priorities-lists resulting from our study is an important contribution to this research and should be used to shape future research efforts.**

 3. The outputs are quite distinct from those of a JLA process - so I suggest the authors refer to the JLA perhaps once, and instead describe their own process and the rationale for how they have approached it, what they expect the impact to be, and their perceived value of their outputs.

 Different does not mean better or worse - this is a different process to the JLA and may have strengths or weaknesses as a result. Perhaps these could be explored in the article. The JLA is not a set of methods, but the principles and values that underpin partnership working are absolutely key to it and these are not described in the approach in this paper and I therefore recommend that the suggestions that this process is linked to the JLA approach are reduced.

**Response: We accept and agree that our process varies from the one of JLA and we have erased the sentence of JLA in the abstract and reframed sentences where we mention JLA  in the method section, as well as reduced the numbers of references to the JLA guidance. We have elaborated on strengths and weaknesses of the current study in the discussion and added some clarifications in the introduction about the differences between our approach and the JLA method.**

**We would like to thank the reviewers for their valuable comments.**
